# 2-(3,4-Dimethyl-5,5-dioxo-2*H*,4*H*-pyrazolo­[4,3-*c*][1,2]benzothia­zin-2-yl)acetic acid

**DOI:** 10.1107/S1600536812023677

**Published:** 2012-05-31

**Authors:** Sana Aslam, Hamid Latif Siddiqui, Matloob Ahmad, Muhammad Zia-ur-Rehman, Masood Parvez

**Affiliations:** a Institute of Chemistry, University of the Punjab, Lahore 54590, Pakistan; b Department of Chemistry, Government College University, Faisalabad 38000, Pakistan; c Applied Chemistry Research Centre, PCSIR Laboratories Complex, Lahore 54600, Pakistan; d Department of Chemistry, The University of Calgary, 2500 University Drive NW, Calgary, Alberta, Canada T2N 1N4

## Abstract

In the title mol­ecule, C_13_H_13_N_3_O_4_S, the heterocyclic thia­zine ring adopts a half-chair conformation in which the S and an adjacent C atom are displaced by 0.919 (3) and 0.300 (4) Å, respectively, on the same side of the mean plane formed by the remaining ring atoms. The mean planes of the benzene and pyrazole rings are inclined at a dihedral angle of 18.32 (12)° with respect to each other. The acetate group is oriented at 80.75 (8)° with respect to the pyrazole ring. The crystal structure is stabilized by O—H⋯N and C—H⋯O hydrogen bonds, resulting in fused eight- and seven-membered rings with *R*_2_^2^(8) and *R*_2_^2^(7) graph-set motifs, respectively.

## Related literature

For the biological activity of benzothia­zine derivatives, see: Turck *et al.* (1996 ▶); Silverstein *et al.* (2000 ▶); Lombardino *et al.* (1973 ▶); Zinnes *et al.* (1973 ▶); Ahmad *et al.* (2010 ▶). For related structures, see: Siddiqui *et al.* (2008 ▶, 2009 ▶). For graph-set notation, see: Bernstein *et al.* (1995 ▶).
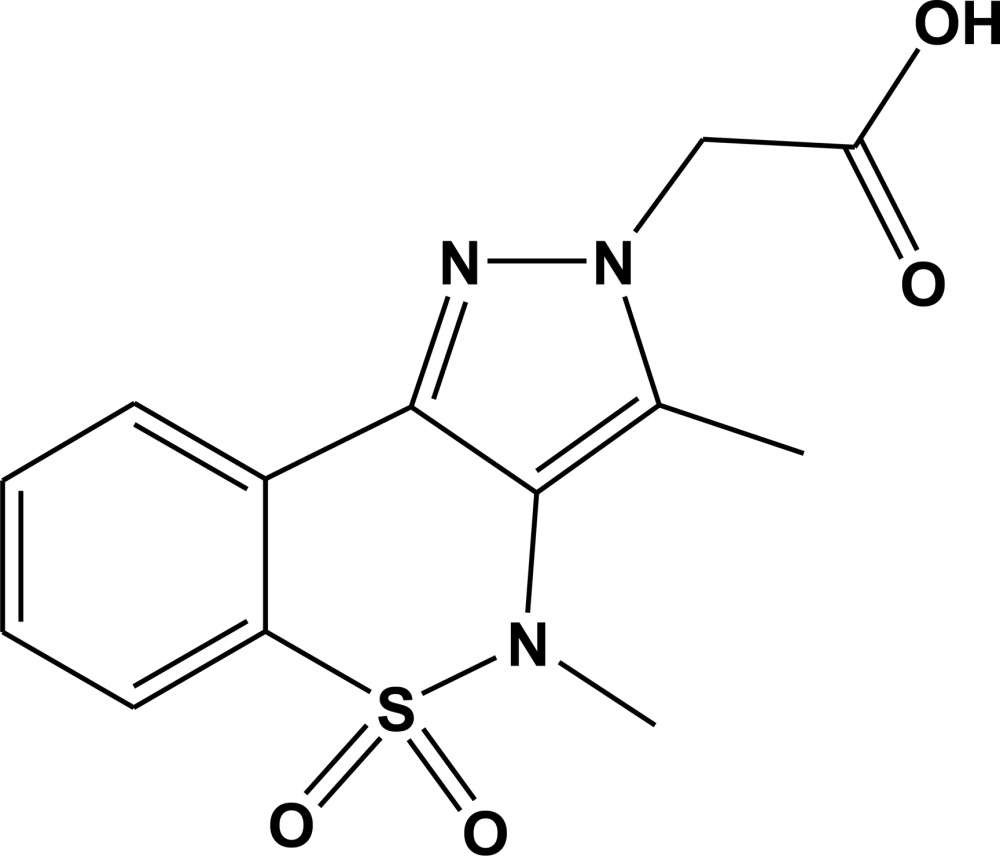


## Experimental

### 

#### Crystal data

C_13_H_13_N_3_O_4_S
*M* *_r_* = 307.32
Monoclinic, 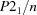

*a* = 10.495 (4) Å
*b* = 8.415 (2) Å
*c* = 15.136 (6) Å
β = 91.034 (19)°
*V* = 1336.5 (8) Å^3^
*Z* = 4
Mo *K*α radiation
μ = 0.26 mm^−1^
*T* = 173 K
0.14 × 0.12 × 0.10 mm

#### Data collection

Nonius KappaCCD diffractometer
Absorption correction: multi-scan (*SORTAV*; Blessing, 1997 ▶) *T*_min_ = 0.964, *T*_max_ = 0.974
5770 measured reflections
3048 independent reflections
2196 reflections with *I* > σ(*I*)
*R*_int_ = 0.033

#### Refinement

*R*[*F* ^2^ > 2σ(*F* ^2^)] = 0.042
*wR*(*F* ^2^) = 0.110
*S* = 1.03
3048 reflections
193 parameters
H-atom parameters constrained
Δρ_max_ = 0.25 e Å^−3^
Δρ_min_ = −0.35 e Å^−3^

### 

Data collection: *COLLECT* (Hooft, 1998 ▶); cell refinement: *DENZO* (Otwinowski & Minor, 1997 ▶); data reduction: *SCALEPACK* (Otwinowski & Minor, 1997 ▶); program(s) used to solve structure: *SHELXS97* (Sheldrick, 2008 ▶); program(s) used to refine structure: *SHELXL97* (Sheldrick, 2008 ▶); molecular graphics: *ORTEP-3 for Windows* (Farrugia, 1997 ▶); software used to prepare material for publication: *SHELXL97*.

## Supplementary Material

Crystal structure: contains datablock(s) global, I. DOI: 10.1107/S1600536812023677/pk2415sup1.cif

Structure factors: contains datablock(s) I. DOI: 10.1107/S1600536812023677/pk2415Isup2.hkl

Supplementary material file. DOI: 10.1107/S1600536812023677/pk2415Isup3.cml

Additional supplementary materials:  crystallographic information; 3D view; checkCIF report

## Figures and Tables

**Table 1:** Hydrogen-bond geometry (Å, °)

*D*—H⋯*A*	*D*—H	H⋯*A*	*D*⋯*A*	*D*—H⋯*A*
O4—H4*O*⋯N2^i^	0.84	1.90	2.724 (2)	165
C9—H9*A*⋯O2^ii^	0.98	2.59	3.297 (3)	129
C5—H5⋯O4^iii^	0.95	2.59	3.476 (3)	155
C12—H12*B*⋯O3^iii^	0.99	2.35	3.303 (3)	160

Symmetry codes: (i) 
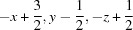
; (ii) 
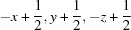
; (iii) 
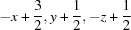
.

**Fig. 1.**
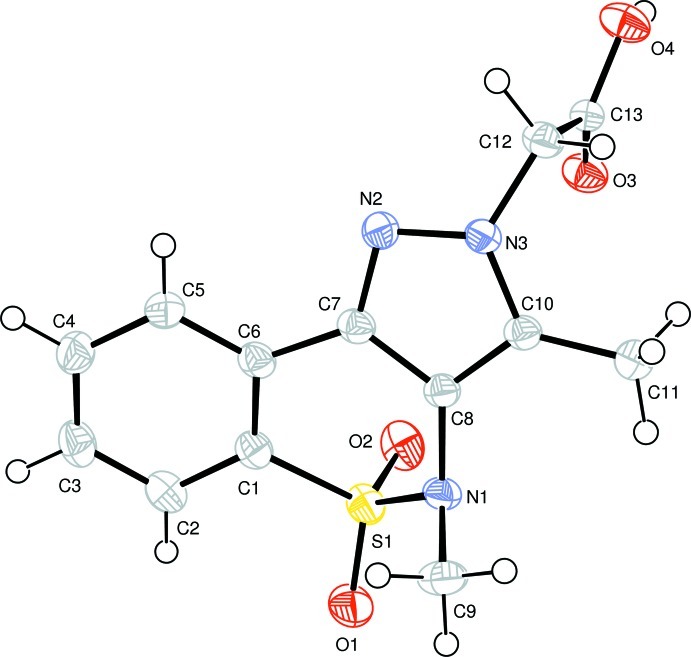
The molecular structure of the title compound with displacement ellipsoids drawn at the 50% probability level. H atoms are presented as small spheres of arbitrary radius.

**Fig. 2.**
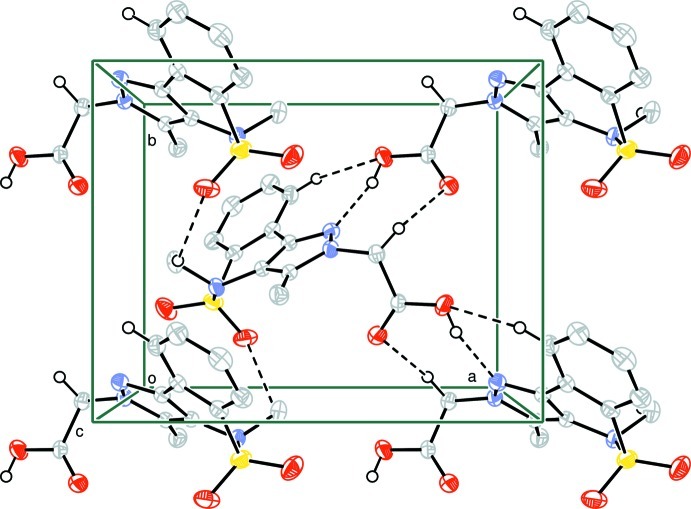
A view of the hydrogen bonding interactions (dotted lines) in the crystal structure of the title compound. H atoms not participating in hydrogen-bonding were omitted for clarity.

## supplementary crystallographic information

### Comment

Meloxicam and Celecoxib are well known for their selective inhibition of the cox-2 enzyme that is responsible for inflammation (Turck *et al.*, 1996; Silverstein *et al.*, 2000). Though a number of benzothiazine based compounds have shown anti-inflammatory and analgesic character, yet there is a huge scope for selective cox-2 inhibitors in this family of heterocyclic compounds (Lombardino *et al.*, 1973; Zinnes *et al.*, 1973). In continuing the pursuit of potential drugs in this category, we have fused benzothiazine and pyrazole heterocycles that are core nuclei of meoxicam and celecoxib, respectively (Ahmad *et al.*, 2010), we have synthesized and determined the crystal structure of the title compound which is presented in this paper.

The bond distances and angles in the title compound (Fig. 1) agree very well with the corresponding bond distances and angles reported in closely related compounds (Siddiqui *et al.*, 2008; 2009). The heterocyclic thiazine ring adopts a twist chair conformation with atoms S1 and C1 displaced by 0.919 (3) and 0.300 (4) Å, respectively, on the same side of the mean plane formed by the remaining ring atoms (r.m.s. deviation 0.012 for N1/C6–C8 atoms). The mean-plane of the benzene ring C1–C6 makes a dihedral angle 18.32 (12)° with the mean-plane of the pyrazolyl ring (N2/N3/C7/C8/C10). The mean-plane of the acetate group (O3/O4/C12/C13) lies at 80.75 (8)° with respect to the pyrazolyl ring. The crystal structure is stabilized by intermolecular hydrogen bonding interactions (Fig. 2 and Table 1). The hydrogen bonds O4—H4O···N2 and C12—H12B···O3 result in eight membered rings with a *R*_2_^2^(8) motif while C5—H5···O4 hydrogen bonding results in a seven membered ring with a *R*_2_^2^(7) motif (Bernstein *et al.*, 1995); both rings are fused together and result in chains of molecules along the *b*-axis in a zigzag fashion. Moreover, C9—H9A···O2 interactions link the title molecules into chains along the *b*-axis further consolidating the crystal packing.

### Experimental

3,4-Dimethyl-2,4-dihydropyrazolo[4,3-*c*][1,2]benzothiazine 5,5-dioxide (5.0 g, 0.020 mole) and bromoacetic acid (3.31 g, 0.024 mole) were dissolved in anhydrous dimethyl formamide (15 ml) and anhydrous potassium carbonate (6.62 g, 0.048 mole) was added to it in small portions. The resulting reaction mixture was stirred for 2.5 h under a nitrogen atmosphere. The contents of the flask were poured over ice cold 10% HCl. Transparent crystals were grown from an aqueous solution, and were used for X-ray crystallographic studies.

### Refinement

All H atoms were positioned geometrically and refined using a riding model, with O—H = 0.84 Å and C—H = 0.95, 0.98 and 0.99 Å, for aryl, methyl and methylene H-atoms, respectively. The *U*_iso_(H) were included at 1.5*U*_eq_(O) or 1.2*U*_eq_(C).

### Crystal data

**Table:**

C_13_H_13_N_3_O_4_S	*F*(000) = 640
*M**_r_* = 307.32	*D*_x_ = 1.527 Mg m^−^^3^
Monoclinic, *P*2_1_/*n*	Mo *K*α radiation, λ = 0.71073 Å
Hall symbol: -P 2yn	Cell parameters from 1947 reflections
*a* = 10.495 (4) Å	θ = 1.0–27.5°
*b* = 8.415 (2) Å	µ = 0.26 mm^−^^1^
*c* = 15.136 (6) Å	*T* = 173 K
β = 91.034 (19)°	Block, colorless
*V* = 1336.5 (8) Å^3^	0.14 × 0.12 × 0.10 mm
*Z* = 4	

### Data collection

**Table:**

Nonius KappaCCD diffractometer	3048 independent reflections
Radiation source: fine-focus sealed tube	2196 reflections with *I* > σ(*I*)
Graphite monochromator	*R*_int_ = 0.033
ω and φ scans	θ_max_ = 27.5°, θ_min_ = 4.1°
Absorption correction: multi-scan (*SORTAV*; Blessing, 1997)	*h* = −13→13
*T*_min_ = 0.964, *T*_max_ = 0.974	*k* = −10→10
5770 measured reflections	*l* = −19→19

### Refinement

**Table:**

Refinement on *F*^2^	Primary atom site location: structure-invariant direct methods
Least-squares matrix: full	Secondary atom site location: difference Fourier map
*R*[*F*^2^ > 2σ(*F*^2^)] = 0.042	Hydrogen site location: difference Fourier map
*wR*(*F*^2^) = 0.110	H-atom parameters constrained
*S* = 1.03	*w* = 1/[σ^2^(*F*_o_^2^) + (0.047*P*)^2^ + 0.650*P*] where *P* = (*F*_o_^2^ + 2*F*_c_^2^)/3
3048 reflections	(Δ/σ)_max_ < 0.001
193 parameters	Δρ_max_ = 0.25 e Å^−^^3^
0 restraints	Δρ_min_ = −0.35 e Å^−^^3^

### Special details

**Table:**

Geometry. All e.s.d.'s (except the e.s.d. in the dihedral angle between two l.s. planes) are estimated using the full covariance matrix. The cell e.s.d.'s are taken into account individually in the estimation of e.s.d.'s in distances, angles and torsion angles; correlations between e.s.d.'s in cell parameters are only used when they are defined by crystal symmetry. An approximate (isotropic) treatment of cell e.s.d.'s is used for estimating e.s.d.'s involving l.s. planes.
Refinement. Refinement of *F*^2^ against ALL reflections. The weighted *R*-factor *wR* and goodness of fit *S* are based on *F*^2^, conventional *R*-factors *R* are based on *F*, with *F* set to zero for negative *F*^2^. The threshold expression of *F*^2^ > σ(*F*^2^) is used only for calculating *R*-factors(gt) *etc*. and is not relevant to the choice of reflections for refinement. *R*-factors based on *F*^2^ are statistically about twice as large as those based on *F*, and *R*- factors based on ALL data will be even larger.

### Fractional atomic coordinates and isotropic or equivalent isotropic displacement parameters (Å^2^)

**Table:**

	*x*	*y*	*z*	*U*_iso_*/*U*_eq_	
S1	0.20345 (5)	0.30572 (6)	0.10421 (3)	0.03013 (16)	
O1	0.07080 (15)	0.28041 (19)	0.08552 (10)	0.0419 (4)	
O2	0.29133 (16)	0.17922 (17)	0.08858 (10)	0.0392 (4)	
O3	0.65265 (14)	0.19936 (17)	0.27745 (10)	0.0328 (4)	
O4	0.82589 (14)	0.30151 (18)	0.34514 (10)	0.0344 (4)	
H4O	0.8579	0.2128	0.3330	0.052*	
N1	0.21936 (16)	0.35819 (19)	0.20921 (10)	0.0258 (4)	
N2	0.52959 (16)	0.54568 (18)	0.19902 (10)	0.0246 (4)	
N3	0.52853 (15)	0.48638 (19)	0.28304 (10)	0.0234 (4)	
C1	0.25731 (19)	0.4733 (2)	0.04545 (12)	0.0261 (4)	
C2	0.1949 (2)	0.5191 (2)	−0.03202 (13)	0.0306 (5)	
H2	0.1192	0.4665	−0.0512	0.037*	
C3	0.2448 (2)	0.6428 (3)	−0.08089 (13)	0.0344 (5)	
H3	0.2035	0.6747	−0.1344	0.041*	
C4	0.3543 (2)	0.7200 (3)	−0.05234 (13)	0.0332 (5)	
H4	0.3889	0.8027	−0.0873	0.040*	
C5	0.4142 (2)	0.6782 (2)	0.02673 (13)	0.0284 (5)	
H5	0.4883	0.7338	0.0464	0.034*	
C6	0.36539 (19)	0.5540 (2)	0.07754 (12)	0.0250 (4)	
C7	0.41474 (19)	0.5083 (2)	0.16483 (12)	0.0238 (4)	
C8	0.34399 (18)	0.4208 (2)	0.22614 (12)	0.0225 (4)	
C9	0.1114 (2)	0.4429 (3)	0.25009 (15)	0.0353 (5)	
H9A	0.1249	0.4473	0.3143	0.042*	
H9B	0.0318	0.3862	0.2366	0.042*	
H9C	0.1061	0.5512	0.2264	0.042*	
C10	0.41904 (18)	0.4087 (2)	0.30145 (12)	0.0228 (4)	
C11	0.3945 (2)	0.3369 (2)	0.38914 (13)	0.0302 (5)	
H11A	0.3108	0.2851	0.3879	0.036*	
H11B	0.3958	0.4202	0.4344	0.036*	
H11C	0.4605	0.2580	0.4030	0.036*	
C12	0.64872 (19)	0.4694 (2)	0.33106 (13)	0.0262 (4)	
H12A	0.6344	0.4829	0.3951	0.031*	
H12B	0.7084	0.5533	0.3121	0.031*	
C13	0.70744 (18)	0.3072 (2)	0.31454 (12)	0.0226 (4)	

### Atomic displacement parameters (Å^2^)

**Table:**

	*U*^11^	*U*^22^	*U*^33^	*U*^12^	*U*^13^	*U*^23^
S1	0.0322 (3)	0.0236 (3)	0.0343 (3)	−0.0031 (2)	−0.0082 (2)	0.0006 (2)
O1	0.0357 (9)	0.0422 (9)	0.0471 (9)	−0.0137 (7)	−0.0147 (7)	0.0061 (7)
O2	0.0499 (11)	0.0234 (8)	0.0441 (9)	0.0048 (7)	−0.0049 (7)	−0.0029 (6)
O3	0.0285 (8)	0.0275 (7)	0.0423 (8)	0.0018 (6)	−0.0052 (7)	−0.0026 (7)
O4	0.0251 (8)	0.0325 (8)	0.0454 (9)	0.0070 (6)	−0.0072 (7)	−0.0052 (7)
N1	0.0196 (9)	0.0262 (8)	0.0315 (9)	−0.0028 (7)	−0.0037 (7)	0.0022 (7)
N2	0.0240 (9)	0.0232 (8)	0.0268 (8)	0.0009 (7)	0.0010 (7)	0.0018 (7)
N3	0.0199 (9)	0.0239 (8)	0.0264 (8)	0.0003 (7)	−0.0019 (6)	0.0023 (7)
C1	0.0269 (12)	0.0238 (10)	0.0275 (10)	0.0046 (8)	0.0004 (8)	−0.0016 (8)
C2	0.0326 (13)	0.0298 (11)	0.0292 (10)	0.0041 (9)	−0.0047 (9)	−0.0052 (9)
C3	0.0406 (14)	0.0390 (12)	0.0234 (9)	0.0077 (10)	−0.0018 (9)	0.0026 (9)
C4	0.0337 (13)	0.0371 (12)	0.0291 (10)	0.0030 (10)	0.0057 (9)	0.0067 (9)
C5	0.0238 (11)	0.0310 (11)	0.0304 (10)	0.0012 (9)	0.0030 (8)	0.0019 (9)
C6	0.0226 (11)	0.0247 (10)	0.0277 (9)	0.0055 (8)	0.0010 (8)	−0.0005 (8)
C7	0.0215 (11)	0.0203 (9)	0.0296 (10)	0.0013 (8)	−0.0011 (8)	−0.0005 (8)
C8	0.0203 (10)	0.0195 (9)	0.0277 (9)	0.0005 (8)	0.0004 (8)	0.0009 (8)
C9	0.0210 (11)	0.0393 (12)	0.0456 (12)	−0.0035 (9)	0.0026 (9)	−0.0003 (10)
C10	0.0204 (10)	0.0198 (9)	0.0284 (9)	0.0013 (8)	0.0006 (8)	0.0006 (8)
C11	0.0279 (12)	0.0313 (11)	0.0313 (10)	−0.0019 (9)	−0.0008 (9)	0.0052 (9)
C12	0.0224 (11)	0.0262 (10)	0.0297 (10)	−0.0013 (8)	−0.0042 (8)	−0.0009 (8)
C13	0.0181 (10)	0.0266 (10)	0.0231 (9)	0.0000 (8)	0.0001 (7)	0.0033 (8)

### Geometric parameters (Å, º)

**Table:**

S1—O2	1.4309 (16)	C3—H3	0.9500
S1—O1	1.4315 (17)	C4—C5	1.387 (3)
S1—N1	1.6550 (18)	C4—H4	0.9500
S1—C1	1.765 (2)	C5—C6	1.401 (3)
O3—C13	1.207 (2)	C5—H5	0.9500
O4—C13	1.320 (2)	C6—C7	1.462 (3)
O4—H4O	0.8400	C7—C8	1.407 (3)
N1—C8	1.429 (2)	C8—C10	1.378 (3)
N1—C9	1.483 (3)	C9—H9A	0.9800
N2—C7	1.341 (2)	C9—H9B	0.9800
N2—N3	1.366 (2)	C9—H9C	0.9800
N3—C10	1.355 (2)	C10—C11	1.485 (3)
N3—C12	1.452 (2)	C11—H11A	0.9800
C1—C2	1.388 (3)	C11—H11B	0.9800
C1—C6	1.401 (3)	C11—H11C	0.9800
C2—C3	1.385 (3)	C12—C13	1.520 (3)
C2—H2	0.9500	C12—H12A	0.9900
C3—C4	1.383 (3)	C12—H12B	0.9900
			
O2—S1—O1	118.95 (10)	C1—C6—C7	117.20 (18)
O2—S1—N1	107.64 (9)	N2—C7—C8	110.47 (17)
O1—S1—N1	108.08 (10)	N2—C7—C6	126.05 (17)
O2—S1—C1	107.32 (10)	C8—C7—C6	123.45 (18)
O1—S1—C1	109.78 (9)	C10—C8—C7	106.48 (17)
N1—S1—C1	104.07 (9)	C10—C8—N1	128.98 (17)
C13—O4—H4O	109.5	C7—C8—N1	124.53 (17)
C8—N1—C9	116.85 (16)	N1—C9—H9A	109.5
C8—N1—S1	110.29 (13)	N1—C9—H9B	109.5
C9—N1—S1	117.66 (13)	H9A—C9—H9B	109.5
C7—N2—N3	104.55 (15)	N1—C9—H9C	109.5
C10—N3—N2	112.87 (15)	H9A—C9—H9C	109.5
C10—N3—C12	125.58 (16)	H9B—C9—H9C	109.5
N2—N3—C12	118.72 (15)	N3—C10—C8	105.57 (16)
C2—C1—C6	121.69 (19)	N3—C10—C11	122.79 (17)
C2—C1—S1	119.80 (16)	C8—C10—C11	131.57 (18)
C6—C1—S1	118.48 (15)	C10—C11—H11A	109.5
C3—C2—C1	118.9 (2)	C10—C11—H11B	109.5
C3—C2—H2	120.6	H11A—C11—H11B	109.5
C1—C2—H2	120.6	C10—C11—H11C	109.5
C4—C3—C2	120.44 (19)	H11A—C11—H11C	109.5
C4—C3—H3	119.8	H11B—C11—H11C	109.5
C2—C3—H3	119.8	N3—C12—C13	110.94 (15)
C3—C4—C5	120.8 (2)	N3—C12—H12A	109.5
C3—C4—H4	119.6	C13—C12—H12A	109.5
C5—C4—H4	119.6	N3—C12—H12B	109.5
C4—C5—C6	119.9 (2)	C13—C12—H12B	109.5
C4—C5—H5	120.1	H12A—C12—H12B	108.0
C6—C5—H5	120.1	O3—C13—O4	125.03 (18)
C5—C6—C1	118.26 (18)	O3—C13—C12	124.07 (17)
C5—C6—C7	124.43 (18)	O4—C13—C12	110.90 (16)
			
O2—S1—N1—C8	64.00 (15)	N3—N2—C7—C6	176.09 (18)
O1—S1—N1—C8	−166.34 (12)	C5—C6—C7—N2	−18.7 (3)
C1—S1—N1—C8	−49.68 (15)	C1—C6—C7—N2	165.16 (18)
O2—S1—N1—C9	−158.49 (15)	C5—C6—C7—C8	159.33 (19)
O1—S1—N1—C9	−28.84 (17)	C1—C6—C7—C8	−16.8 (3)
C1—S1—N1—C9	87.83 (16)	N2—C7—C8—C10	1.5 (2)
C7—N2—N3—C10	2.1 (2)	C6—C7—C8—C10	−176.80 (17)
C7—N2—N3—C12	164.15 (16)	N2—C7—C8—N1	−177.84 (17)
O2—S1—C1—C2	105.04 (18)	C6—C7—C8—N1	3.8 (3)
O1—S1—C1—C2	−25.6 (2)	C9—N1—C8—C10	76.3 (3)
N1—S1—C1—C2	−141.05 (16)	S1—N1—C8—C10	−145.81 (18)
O2—S1—C1—C6	−73.08 (18)	C9—N1—C8—C7	−104.5 (2)
O1—S1—C1—C6	156.31 (16)	S1—N1—C8—C7	33.4 (2)
N1—S1—C1—C6	40.84 (18)	N2—N3—C10—C8	−1.2 (2)
C6—C1—C2—C3	3.3 (3)	C12—N3—C10—C8	−161.78 (17)
S1—C1—C2—C3	−174.78 (15)	N2—N3—C10—C11	−178.68 (17)
C1—C2—C3—C4	−0.6 (3)	C12—N3—C10—C11	20.8 (3)
C2—C3—C4—C5	−1.8 (3)	C7—C8—C10—N3	−0.2 (2)
C3—C4—C5—C6	1.5 (3)	N1—C8—C10—N3	179.14 (18)
C4—C5—C6—C1	1.1 (3)	C7—C8—C10—C11	177.0 (2)
C4—C5—C6—C7	−175.00 (19)	N1—C8—C10—C11	−3.7 (4)
C2—C1—C6—C5	−3.5 (3)	C10—N3—C12—C13	70.0 (2)
S1—C1—C6—C5	174.58 (15)	N2—N3—C12—C13	−89.5 (2)
C2—C1—C6—C7	172.87 (18)	N3—C12—C13—O3	−10.2 (3)
S1—C1—C6—C7	−9.0 (2)	N3—C12—C13—O4	168.99 (16)
N3—N2—C7—C8	−2.2 (2)		

### Hydrogen-bond geometry (Å, º)

**Table:**

*D*—H···*A*	*D*—H	H···*A*	*D*···*A*	*D*—H···*A*
O4—H4*O*···N2^i^	0.84	1.90	2.724 (2)	165
C9—H9*A*···O2^ii^	0.98	2.59	3.297 (3)	129
C5—H5···O4^iii^	0.95	2.59	3.476 (3)	155
C12—H12*B*···O3^iii^	0.99	2.35	3.303 (3)	160
C9—H9*B*···O1	0.98	2.49	2.867 (3)	102

Symmetry codes: (i) −*x*+3/2, *y*−1/2, −*z*+1/2; (ii) −*x*+1/2, *y*+1/2, −*z*+1/2; (iii) −*x*+3/2, *y*+1/2, −*z*+1/2.
